# Perceived Benefits and Implementation Factors of a Multicomponent Exercise Program in Care Facilities in Hong Kong

**DOI:** 10.1093/geroni/igaf122.3635

**Published:** 2025-12-31

**Authors:** Kin Tung Chan, Chi Tat Fung, Ziwei Zeng, Yijian Yang

**Affiliations:** Hong Kong Sheng Kung Hui Multi-disciplinary Outreaching Support Teams for the Elderly (Kowloon Central Cluster), Hong Kong, China (People’s Republic); Hong Kong Sheng Kung Hui Multi-disciplinary Outreaching Support Teams for the Elderly (Kowloon Central Cluster), Hong Kong, China (People’s Republic); The Chinese University of Hong Kong, Hong Kong, China (People’s Republic); The Chinese University of Hong Kong, Hong Kong, China (People’s Republic)

## Abstract

Older adults in long-term care (LTC) face accelerated functional decline and high fall risk, traditional prevention programs often overlook upper-limb and core strengthening, which is important for safe mobility. Mobility-Fit, a multicomponent exercise program, was developed to address these gaps in care facilities. This study explored perceived benefits and factors influencing program implementation through semi-structured interviews with 21 LTC residents (13 intervention, 8 control) and 4 care staffs following a cluster randomized controlled trial. Participants reported enhanced health-related quality of life, increased confidence, and enjoyment, with 76.9% describing the program as novel and beneficial. Nearly half (46.2%) highlighted meaningful outcomes such as mutual respect, personalized suitability, and enjoyment from group-based exercise. Additionally, 38.5% of participants in the intervention group reported no fear of falling, compared to 25% in the control group. Key facilitators included tailored exercise adaptations, professional supervision, supportive management by the care professional team. The use of familiar music and culturally resonant exercise incorporating simple equipment such as TheraBands and fitness balls, further enhanced engagement among participants. Challenges included poor vision, limited mobility, and fluctuating health among residents, with difficulties in maintaining engagement and adapting content for diverse functional levels. Nevertheless, staffs emphasized that adaptive strategies and collaborative problem-solving supported program feasibility. Involving family members and integrating culturally relevant elements proved effective. Mobility-Fit was perceived as safe, enjoyable, and feasible when tailored to residents’ capacities and cultural context. Our findings support integrating culturally adapted exercise programs in LTC settings to promote physical and psychosocial well-being of older adults.

